# Retraction Note: METTL3 improves cardiomyocyte proliferation upon myocardial infarction via upregulating miR-17-3p in a DGCR8-dependent manner

**DOI:** 10.1038/s41420-025-02344-9

**Published:** 2025-02-13

**Authors:** Kun Zhao, Chuanxi Yang, Jing Zhang, Wei Sun, Bin Zhou, Xiangqing Kong, Jing Shi

**Affiliations:** 1 Department of Cardiology, The First Affiliated Hospital of Nanjing Medical University, Nanjing, 210029 Jiangsu China; 2 Department of Cardiology, Yangpu Hospital, Tongji University School of Medicine, Shanghai, 200082 China; 3 Departments of Genetics, Pediatrics, and Medicine (Cardiology), Wilf Cardiovascular Research Institute, Albert Einstein College of Medicine, Bronx, NY 10461 USA

Retraction to: *Cell Death Discovery* 10.1038/s41420-021-00688-6, published online 13 October 2021

The Editors-in-Chief have retracted this article. A number of image integrity concerns were raised, including apparent overlap in panels showing different experimental conditions. Specifically:

- partial overlap between panels in figures 1B and 1C,
- re-use of an image with different data label in figures 2C and 7D,
- additional concerns were found between figures 3C and 7D,
- partial overlap was found within figure 4 between two images,
- re-use of an image with different data label in figure 4,
- two apparent duplications across 5F in this paper and figure 8A in another paper by the same authors [1].

The Editors-in-Chief have therefore lost confidence in the data supporting the conclusions of this article.

Author Shi Jing has not explicitly stated if they agree or disagree with this retraction. All other authors have not responded to correspondence regarding this retraction.
